# Flexibility of brain dynamics is increased and predicts clinical impairment in relapsing–remitting but not in secondary progressive multiple sclerosis

**DOI:** 10.1093/braincomms/fcae112

**Published:** 2024-04-02

**Authors:** Lorenzo Cipriano, Roberta Minino, Marianna Liparoti, Arianna Polverino, Antonella Romano, Simona Bonavita, Maria Agnese Pirozzi, Mario Quarantelli, Viktor Jirsa, Giuseppe Sorrentino, Pierpaolo Sorrentino, Emahnuel Troisi Lopez

**Affiliations:** Department of Medical, Motor and Wellness Sciences, University of Naples ‘Parthenope’, 80133 Naples, Italy; Department of Medical, Motor and Wellness Sciences, University of Naples ‘Parthenope’, 80133 Naples, Italy; Department of Philosophical, Pedagogical and Quantitative-Economic Sciences, University of Chieti-Pescara ‘G. d’Annunzio’, 66100 Chieti, Italy; Institute of Diagnosis and Therapy Hermitage Capodimonte, 80145 Naples, Italy; Department of Medical, Motor and Wellness Sciences, University of Naples ‘Parthenope’, 80133 Naples, Italy; Department of Advanced Medical and Surgical Sciences, University of Campania ‘L. Vanvitelli’, 81100 Naples, Italy; Department of Advanced Medical and Surgical Sciences, University of Campania ‘L. Vanvitelli’, 81100 Naples, Italy; Biostructure and Bioimaging Institute, CNR, 80145 Naples, Italy; Institut de Neurosciences des Systèmes, Inserm, INS, Aix-Marseille University, 13005 Marseille, France; Department of Medical, Motor and Wellness Sciences, University of Naples ‘Parthenope’, 80133 Naples, Italy; Institute of Diagnosis and Therapy Hermitage Capodimonte, 80145 Naples, Italy; Institute of Applied Sciences and Intelligent Systems, National Research Council, 80078 Pozzuoli, Italy; Institut de Neurosciences des Systèmes, Inserm, INS, Aix-Marseille University, 13005 Marseille, France; Department of Biomedical Sciences, University of Sassari, 07100 Sassari, Italy; Institute of Applied Sciences and Intelligent Systems, National Research Council, 80078 Pozzuoli, Italy

**Keywords:** magnetoencephalography, neuronal avalanches, brain flexibility, inflammation, neurodegeneration

## Abstract

Large-scale brain activity has long been investigated under the erroneous assumption of stationarity. Nowadays, we know that resting-state functional connectivity is characterized by aperiodic, scale-free bursts of activity (i.e. neuronal avalanches) that intermittently recruit different brain regions. These different patterns of activity represent a measure of brain flexibility, whose reduction has been found to predict clinical impairment in multiple neurodegenerative diseases such as Parkinson’s disease, amyotrophic lateral sclerosis and Alzheimer’s disease. Brain flexibility has been recently found increased in multiple sclerosis, but its relationship with clinical disability remains elusive. Also, potential differences in brain dynamics according to the multiple sclerosis clinical phenotypes remain unexplored so far. We performed a brain dynamics study quantifying brain flexibility utilizing the ‘functional repertoire’ (i.e. the number of configurations of active brain areas) through source reconstruction of magnetoencephalography signals in a cohort of 25 multiple sclerosis patients (10 relapsing–remitting multiple sclerosis and 15 secondary progressive multiple sclerosis) and 25 healthy controls. Multiple sclerosis patients showed a greater number of unique reconfigurations at fast time scales as compared with healthy controls. This difference was mainly driven by the relapsing–remitting multiple sclerosis phenotype, whereas no significant differences in brain dynamics were found between secondary progressive multiple sclerosis and healthy controls. Brain flexibility also showed a different predictive power on clinical disability according to the multiple sclerosis type. For the first time, we investigated brain dynamics in multiple sclerosis patients through high temporal resolution techniques, unveiling differences in brain flexibility according to the multiple sclerosis phenotype and its relationship with clinical disability.

## Teaser

Cipriano *et al.* studied the brain flexibility in individuals with relapsing–remitting and secondary progressive multiple sclerosis, through source-reconstructed magnetoencephalography data. Brain flexibility was greater than controls only in relapsing–remitting patients. This study unveils differences in brain dynamics according to the multiple sclerosis phenotype and its relationship with clinical disability.

## Graphical Abstract

**Graphical Abstract:**
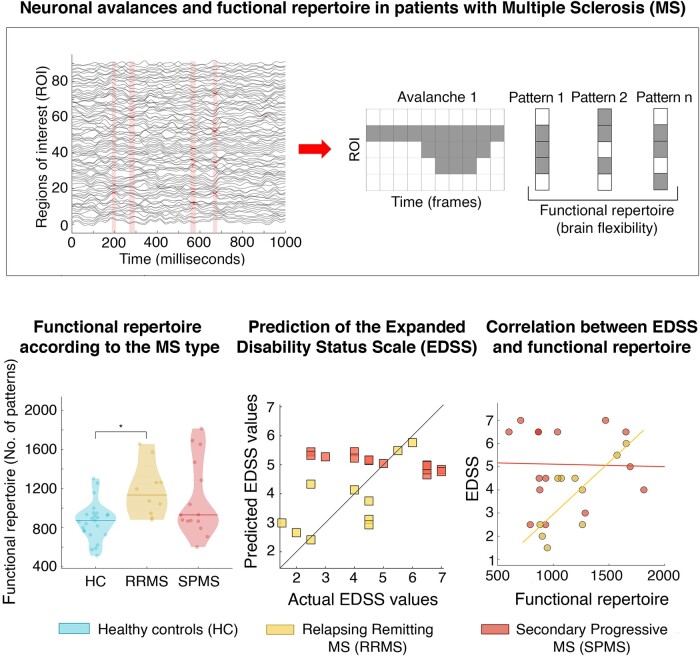

## Introduction

Multiple sclerosis (MS) is a chronic inflammatory disease of the central nervous system characterized by a complex association of both demyelination and diffuse neurodegeneration of the grey and white matter.^1^ MS occurs most often with a clinical phenotype characterized by a relapsing–remitting course (RRMS). However, around 50% of MS patients can evolve to a secondary progressive form (SPMS), and a minority may show worsening from the onset, the primary progressive form (PPMS).^1,2^ Recent studies support the idea that RRMS and SPMS are part of a disease continuum, in which the phase transition is driven by the change in the balance between inflammatory and neurodegenerative mechanisms.^3^ The different clinical presentations, which are a consequence of distinct underlying pathophysiological mechanisms, may also explain the large variability of responses to the currently available immunosuppressive and immunomodulatory treatments.^1,3,4^

MRI is currently established as a key diagnostic tool in MS^5^ due to its ability to detect the spatial and temporal distribution of MS-associated lesions. Nevertheless, years of use of MRI have shown that only a small fraction of MS clinical features and outcomes can be explained by the lesion load. This mismatch is called the clinical–radiological paradox and highlights our lack of understanding of this complex disease.^6^

Many functional MRI (fMRI) studies deployed neural network theory in the attempt to shed light on the functional effects of the neuropathological processes that characterize the different clinical phenotypes. Taken together, these studies demonstrated relationships between changes in functional connectivity (FC) among specific brain regions and clinical features of the disease.^7-9^ However, these results were internally inconsistent, as they often failed to replicate. For instance, some studies found that increased resting-state FC (RS-FC) of selected brain regions/networks was related to better cognitive performances,^7,10^ while others showed an increased RS-FC of the same regions/networks in cognitively impaired individuals.^8,9^

Potential explanations for these discrepancies include the temporal neuropathological evolution of the disease (i.e. different studies were performed in different disease stages), its clinical heterogeneity, the methodological differences across fMRI studies and, finally, the intrinsic limitations of the fMRI, including its low temporal resolution. The low temporal resolution represents a weak point of the fMRI, because the temporal smoothing induced by the slow haemodynamic response reduces the ability to evaluate brain reconfigurations at fast time scales. Conversely, magneto- and encephalography (MEG, EEG) provides a more direct assessment of the brain’s fast activities.^11^

Large-scale brain scans have been processed typically under the assumption of stationarity. However, now we know that RS-FC evolves over time in a non-linear fashion.^12^ In particular, brain activity is characterized by aperiodic, scale-free bursts of activity (i.e. neuronal avalanches) that intermittently interconnect brain regions^13-15^ and that account for most of the time-averaged FC.^16^ In particular, aperiodic bursts reconfigure over time, giving rise to rich, non-stereotyped dynamics. In fact, healthy brains constantly recruit different brain regions, generating a high number of patterns of activations. Thus, the number of such patterns represents a measure of brain flexibility dynamics, whose reduction has been found to predict clinical impairment in multiple neurodegenerative diseases.^17,18^

Based on our previous observations about brain dynamics in neurodegenerative diseases such as probable Alzheimer’s disease,^19^ Parkinson’s disease^17^ and amyotrophic lateral sclerosis,^18^ in the present work, we hypothesize that MS could also be characterized by variations in brain flexibility, which would be related to, and predictive of, the subject-specific clinical impairment. We also wondered whether the underlying disease mechanisms that characterize the two distinct forms of MS (relapsing–remitting and progressive) could reflect itself in different brain dynamics and if this difference could help in predicting clinical disability.

To test these hypotheses, we source-reconstructed MEG scans performed in 25 MS patients (10 RRMS and 15 SPMS) and 25 healthy controls (HC). To estimate flexibility, we calculated the number of unique patterns of neuronal avalanches expressed in each MEG recording. Operationally, a neuronal avalanche is defined as an event starting when at least one brain region deviates from its baseline activity and ending when all regions return to their normal level of activity. An avalanche pattern is defined as the set of all the brain areas that were recruited at any moment during an avalanche. The functional repertoire is defined as the set of the unique patterns that occurred over time, and its size can be seen as a surrogate marker of brain flexibility. Finally, we implemented a multilinear regression model with ‘k-fold cross-validation’ to verify the ability of the size of the functional repertoire to predict, at individual level, the clinical impairment assessed by the Expanded Disability Status Scale (EDSS).

## Materials and methods

### Participants

Twenty-five MS patients (7 males and 18 females) and 25 age-, sex- and education-matched HC were recruited from 1 April 2018 to 9 November 2018. MS was diagnosed in accordance with the 2017 revision of the McDonald criteria.^5^ MS individuals were further classified in RRMS and SPMS. The eligibility of the patients was defined according to the following exclusion criteria: (i) use of illicit drugs, stimulants, amphetamines, barbiturates and cannabis; (ii) a history of CNS disorder other than MS; (iii) severe mental illness; and (iv) other systemic disorders with possible secondary involvement of the CNS.

MS patients underwent a clinical examination performed by an experienced neurologist. The EDSS^20^ was used to evaluate disease-related disability. Fatigue was assessed by the Fatigue Severity Scale (FSS),^21^ while neurocognitive function was evaluated by the Symbol Digit Modalities Test (SDMT).^22^ Key symptoms of depression were studied through the Beck Depression Inventory (BDI) self-rated scale.^23^ The study protocol was approved by the Local Ethics Committee (ASL-NA1) with protocol number Prot.n.93C.E./Reg. n.14-17OSS. All procedures performed were in accordance with the ethical standards of the institutional research committee, and all participants provided written informed consent in accordance with the Declaration of Helsinki.

### MEG and MRI acquisition, preprocessing, and source reconstruction

**Figure 1:**
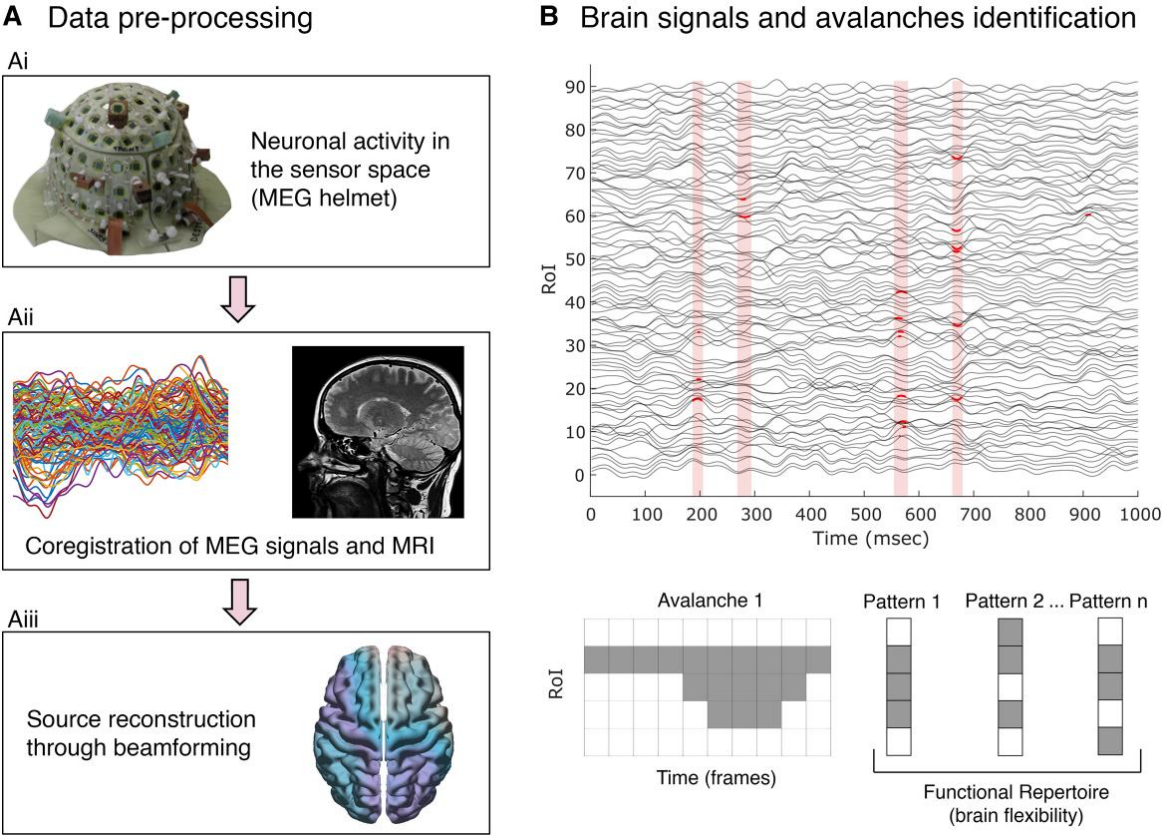
**Pipeline overview and neuronal avalanche representation**. (**A: Ai**) Registration of neuronal activity through MEG. (**Aii**) Cleaned sensor signals (without physiological artefacts) coregistered with structural MRI of each participant. (**Aiii**) Through a beamformer algorithm, the time series of the sources were estimated in ROIs within the brain according to a parcellation based on the AAL atlas. (**B**) The highlighted boxes represent the time frame in which a neuronal avalanche occurred. Specifically, the bold dots indicate the frame in which the time series was above threshold (*Z*-score > 3). In the bottom section of the panel, a schematic representation of a neuronal avalanche is depicted.

MRI and MEG acquisition, preprocessing, source reconstruction and connectivity estimation (Fig. 1A) have been performed according to our previous studies.^24-29^ In particular, patients and HC underwent MRI recorded by a 1.5 T GE Medical System (GE Healthcare, Milwaukee, MI, USA) scanner, obtaining T_1_-weighted images for MEG data coregistration. Three-dimensional high-resolution T_1_-weighted (3D-T_1_) inversion recovery prepared fast spoiled gradient recalled sequence [IR-FSPGR, repetition time (TR) = 8.216 ms, T_1_ = 450 ms, echo time (TE) = 3.08 ms, flip angle = 12, voxel size = 1 × 1 × 1.2 mm^3^) was acquired in 23 out of 25 patients in order to extract volumetric data. The 3D images were processed on MATLAB version R2022a (The MathWorks, Natick, USA) using Statistical Parametric Mapping 12 (SPM12) (Wellcome Trust Centre for Neuroimaging, London, UK; www.fil.ion.ucl.ac.uk/spm). Following lesion detection by the Lesion Segmentation Tool (https://www.applied-statistics.de/lst.html), lesions filling and brain segmentation into GM, WM and CSF has been performed through the segmentation tool provided by the Computational Anatomy Toolbox 12 (CAT12). The Automated Anatomical Labelling (AAL) atlas^30^ has been then applied to normalized segmented brain tissue maps to extract volumetric information of regions of interest (ROIs) labelled according to the same atlas used in the MEG analysis (see below). To this end, normalization of the brains to the Montreal Neurological Institute (MNI) template space was performed using Diffeomorphic Anatomical Registration Through Exponentiated Lie algebra (DARTEL).^31^ The normalized images of each tissue were modulated in order to preserve regional and global volumes. The volumes were exported for external statistical analysis on the RStudio platform (RStudio Team, 2021, Boston, MA; http://www.rstudio.com/).

Concerning the MEG acquisition, preprocessing and source reconstruction, data were acquired using a MEG system composed of 154 magnetometers Superconductive Quantum Interference Device (SQUID) and 9 reference sensors.^32^ The acquisition took place in a magnetically shielded room (ATB, Biomag, ULM, Germany) to reduce external noise. Two consecutive, resting-state, closed eyes, 3.5 min-long recordings, separated by a roughly 2 min-long break, were acquired. The length of the recording was a trade-off between the need to have enough cleaned temporal series and avoid drowsiness.^33,34^ ECG and electro-oculography (EOG) were also coregistered during the scan. Data were sampled at 1024 Hz after antialiasing filtering. Preprocessing and source reconstruction were performed by filtering the MEG data in the 0.5–48 Hz range by applying a fourth-order Butterworth IIR band-pass filter using the FieldTrip toolbox in MATLAB.^35^ After that, a principal component analysis (PCA)^36^ was performed to orthogonalize signals with respect to the reference signals to reduce environmental noise. Independent component analysis (ICA)^37^ was used to remove any ECG and EOG artefacts. MEG data were coregistered with the native MR T_1_-weighted images of each subject. We extracted the time series of 116 AAL-derived ROIs exploiting the volume conduction model introduced by Nolte^38^ and applying the linearly constrained minimum variance^39^ beamformer algorithm in the FieldTrip toolbox.^35^ Finally, we removed the cerebellar ROIs due to poor reliability. Hence, 90 ROIs were used for further analyses. For one HC that refused to undergo MRI, a standard template was used to reconstruct the time series of specific ROIs.

### Analysis of brain dynamics

#### Neuronal avalanches and branching parameter

To quantify the spatiotemporal fluctuations of brain activity, we estimate neuronal avalanches (Fig. 1B). A single neuronal avalanche is defined as an event starting with a fluctuation of the regional brain activity in at least one ROI and ending with the return of all the involved ROIs to their normal activity.^40^

Each of the source-reconstructed signals (derived from the 90 ROIs) was *Z*-transformed and thresholded according to a cut-off of 3 SDs (i.e. *z* > |3|).^17^ A confirmation of the results’ independence from the chosen threshold was performed by changing the threshold from 2.5 to 3.5.

To capture the critical dynamics, we binned the time series^17^ by estimating the suitable time bin length by computing the branching ratio *σ* for each individual, for each avalanche and for each time bin duration.^13^ Specifically, the branching ratio was calculated as follows:


$$\sigma_{i}=\;\overset{N_{\mathrm{bin}}-1}{\underset{\;j=1}{\prod }}\;{\left(\frac{n_{\mathrm{events}}\;(\;j+1)}{n_{\mathrm{events}}\;(j)}\right)}^{\frac{1}{N_{\mathrm{bin}}-1}}\;$$

where σ is the branching parameter of the *i*-th avalanche in the data set, *N*_bin_ is the total number of bins in the *i*-th avalanche and *n*_events_  *j* is the total number of events in the *j*-th bin. After that, we geometrically averaged the results over all avalanches as follows:


$$\sigma =\;\overset{N_{\mathrm{aval}}}{\underset{i=1}{\prod }}\;(\sigma_{i}{)}^{\frac{1}{N_{\mathrm{aval}}}}\;\;\;\;$$

Critical processes are represented by σ = 1 that was present at bin length = 3. However, we repeated our analysis varying time bins from 1 to 5 and obtained similar results. Each avalanche had an avalanche pattern defined as the set of all ROIs that were above the threshold.

#### Functional repertoire

For each individual, we calculated the functional repertoire as the number of unique avalanche patterns expressed during the recording.^17^ Unique indicates that each avalanche pattern is counted only once over the extent of the functional repertoire (i.e. repetitions are discarded).

### Multilinear regression analysis

Starting from the assumption that fluctuations in brain dynamics could predict clinical impairment, we performed a multilinear regression model. The latter was performed by including a clinical feature (EDSS, FSS, SDMT, or BDI) as a dependent variable and MS type and functional repertoire as independent variables. Multicollinearity was assessed through the variance inflation factor (VIF). To validate our approach, we performed *k*-fold cross-validation, with *k* = 5.^41^ Specifically, *k* iterations were performed to train our model and at each iteration, and the *k^th^* subgroup was used as a test set.

### Statistical analysis

Statistical analysis was carried out in MATLAB 2021a and R Studio (http://www.rstudio.com/). A *t*-test and a χ^2^ were used to compare patients and controls for age, educational level and sex. A Wilcoxon rank sum test was used to compare HC and MS groups. The Kruskal–Wallis test was performed to compare HC, RRMS and SPMS groups. The results were corrected by the false discovery rate, and the significance level was set at *P*-value < 0.05. The relationship between the size of the functional repertoire and the clinical scores was investigated in the MS group using the Spearman’s correlation coefficient. The predictive power of the flexibility parameter on clinical features has been investigated through a multilinear regression model.

## Results

### Cohort characteristics

Sociodemographic and clinical characteristics of our cohort (as also reported in Cipriano *et al*.^24^) are reported in Table 1 (and Supplementary Table 1).

**Table 1:** Sociodemographic and clinical characteristics of the cohort

Parameters	MS patients (*n* = 25), mean (±SD)	HC (*n* = 25), mean (±SD)	*P*-values
**Demographic data**			
** Age**	45.68 ± 9.47	45.8 ± 11.83	ns
** Male/female**	7/18	7/18	ns
** Education (years)**	13.36 ± 4.28	13.93 ± 3.91	ns
**MS-specific clinical characteristics**			
	RRMS	SPMS	
** MS phenotype (*n*)**	10	15	
** DD (months ± SD)**	101.20 ± 75.61	221.27 ± 161.78	0.020
** EDSS (mean)**	3.75	5.10	ns
** EDSS (range)**	1.5–6	2.5–7	
** FSS (mean ± SD)**	29.80 ± 13.54	42.80 ± 12.45	0.025
** FSS (range)**	9–49	19–59	
** BDI (mean ± SD)**	11.70 ± 6.70	10.87 ± 10.11	ns
** BDI (range)**	0–19	0–36	
** SDMT (mean ± SD)**	41.80 ± 13.93	35.00 ± 13.59	ns
** SDMT (range)**	15–59	11–58	
** LL**	10.85 ± 17.14	19.55 ± 18.14	0.03
** GM**	666.07 ± 96.14	596.54 ± 72.30	ns
** WM**	346.56 ± 54.04	330.66 ± 70.84	ns

*P*-value from Wilcoxon–Mann–Whitney or Student’s *t*-test, according to the sample data distribution checked with the Kolmogorov–Smirnov test. No significant difference in age, gender and education was found between the two groups. BDI, Beck Depression Inventory; DD, disease duration; EDSS, Expanded Disability Status Scale; FSS, Fatigue Severity Scale; GM, grey matter; HC, healthy controls; LL, lesion load; MS, multiple sclerosis; RRMS, relapsing–remitting MS; SDMT, Symbol Digit Modalities Test; SPMS, secondary progressive MS; WM, white matter. ROI volumes in the SPMS group were available only for 13 patients. Volumes of each ROI are provided in the supplementary materials (Supplementary Table 1). Volumes are defined in mL.

### Analysis of brain dynamics: the functional repertoire

**Figure 2:**
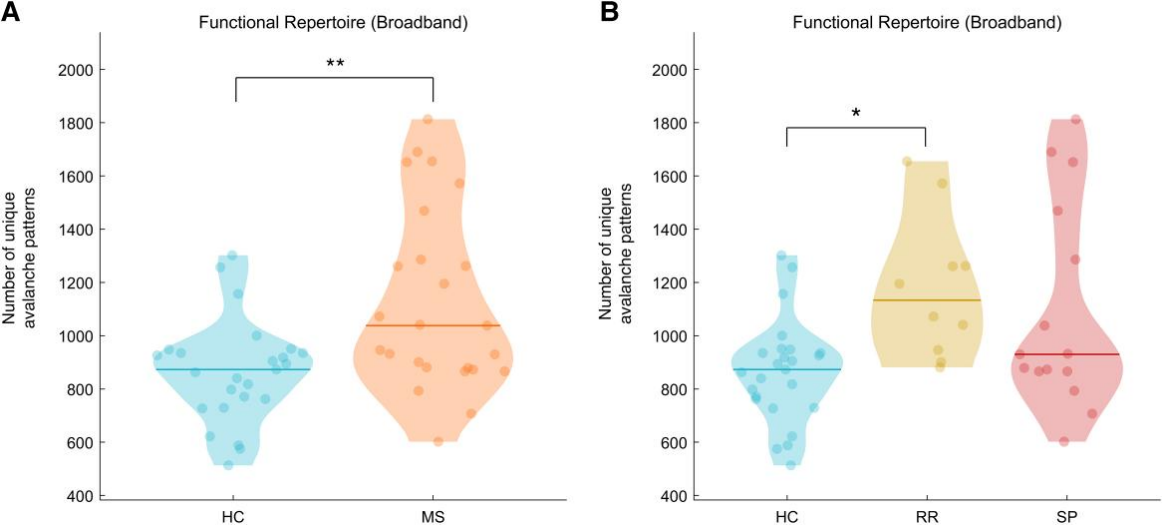
**Brain flexibility comparison**. (**A**) Violin plots (HC in blue and individuals with MS in orange) of the number of unique avalanche patterns (i.e. functional repertoire). (**B**) Violin plots including MS type separation (RRMS in yellow and SPMS in red). The dots in the violins represent the size of the functional repertoire of each individual. The horizontal lines in the violins represent the mean value of each group. Significant *P*-values after *t*-test (**A**) and Kruskal–Wallis test (**B**), respectively: *P < 0.05, **P < 0.01, ***P < 0.001.

The comparison between MS and HC showed a larger functional repertoire in MS patients (*P* = 0.006) (Fig. 2A). We also performed a Kruskal–Wallis test to evaluate differences in the size of the functional repertoire according to the MS clinical form (χ^2^(2) = 9.8, *P* = 0.007) (RRMS and SPMS). As shown in Fig. 2B, the difference in brain flexibility between MS and HC was primarily driven by the RRMS patients (*P* = 0.02).

### Impairment prediction according to MS type

**Figure 3:**
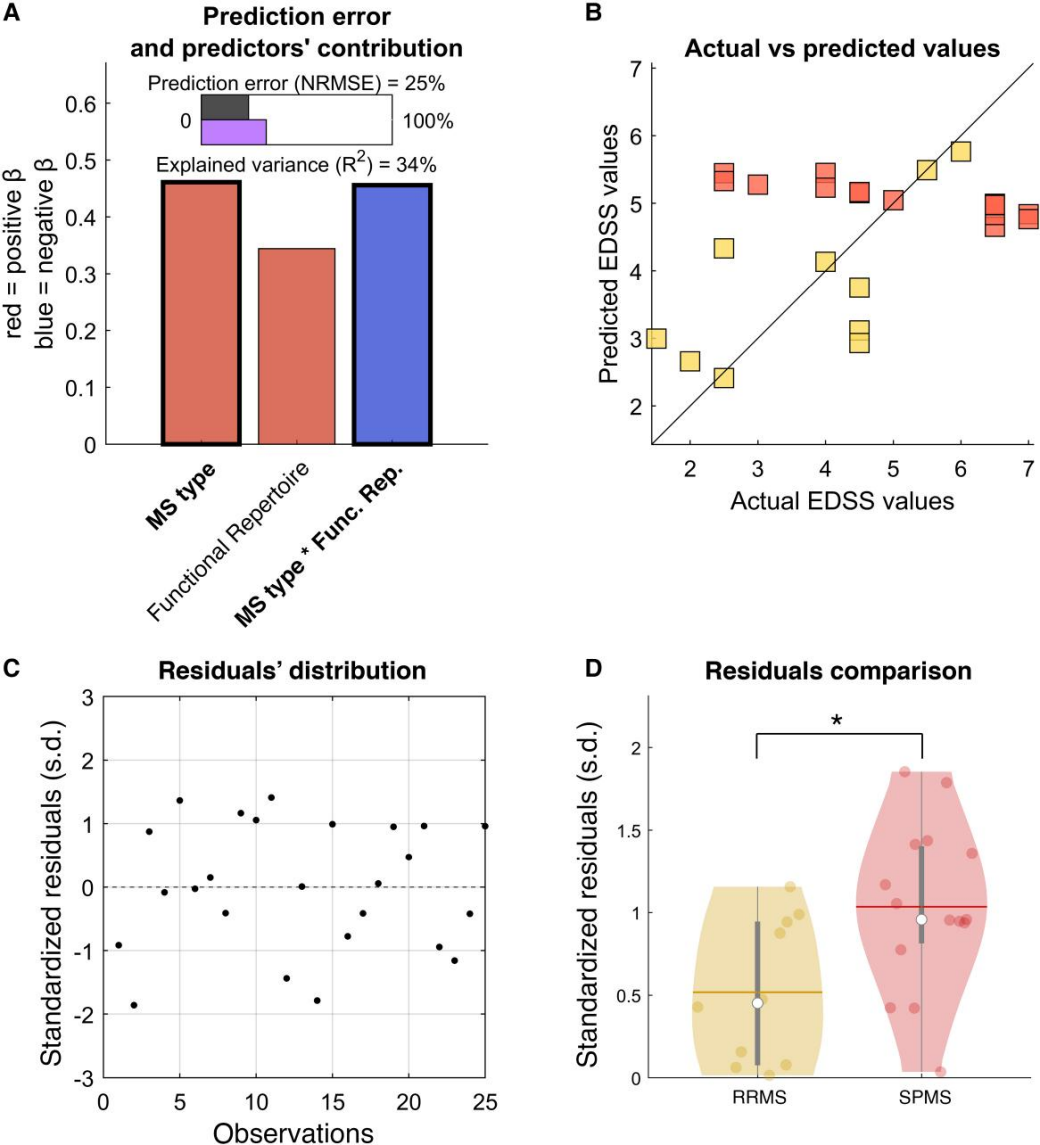
**Clinical impairment prediction**. Multilinear regression analysis with *k-fold* cross-validation was performed to verify the ability of the size of the functional repertoire to predict clinical impairment assessed by the EDSS. The clinical phenotype of MS was added as a predictor into the model (individually and as an interaction with the functional repertoire), to verify whether the prediction would depend upon the MS form (RRMS or SPMS). (**A**) Regression analysis data: *F*-test (*F*(3,21) = 3.61, *P* = 0.03) *R*^2^ = 0.34, normalized root mean square error (NRMSE) = 0.25; significant predictors in bold (MS type, β = 0.461, *P* = 0.018; MS type * functional repertoire, β = −0.456, *P* = 0.0419). (**B**) Scatter plot to compare actual EDSS values with the EDSS values predicted through cross-validation. Since the MS type and the interaction between the MS type and functional repertoire were significant, we used different colours to represent RRMS (yellow) and SPMS (red) and observe the predictions independently. (**C**) Scatter plot of the residual’s distribution. (**D**) Statistical comparison (permutation test with 10,000 iterations) between RRMS and SPMS absolute value of the residuals. Significant lower residuals indicate better prediction of the EDSS values of the RRMS group. *P-value < 0.05. Abbreviations: s.d., standard deviations.

We performed a k-fold cross-validated multilinear regression analysis setting the clinical variables (FSS, EDSS, SDMT, BDI) as dependent variables and tried to predict them by the means of the size of the functional repertoire. We also included the MS phenotype as a further predictor and its interaction with the size of the functional repertoire to account for a possible different behaviour in the relationship between the functional repertoire and the EDSS score. We obtained a significant regression model (*F*(3,21) = 3.61, *P* = 0.03) able to predict 34% of the EDSS variance, with a normalized root mean squared error of the prediction equal to 25%. The significant contribution was determined by the MS type and its interaction with the size of the functional repertoire (beta coefficient = 0.461, *P* = 0.018, and beta coefficient = −0.456, *P* = 0.0419, respectively) (Fig. 3A). The interaction effect between MS type and functional repertoire suggests that the MS type is worthy in obtaining a significant prediction. Indeed, when removing the interaction effect, the model was not predictive anymore, while the MS type only, as a categorical variable, was not enough to predict the EDSS score. Given the MS type relevance, we observed the comparison between actual and predicted EDSS in each group separately (Fig. 3B). It can be noticed that while the predicted values of the RRMS group (yellow) showed a direct agreement with the actual values, this was not the case with the SPMS predictions (red). The distribution of the standardized residuals was then observed (Fig. 3C), and its absolute value was compared between RRMS and SPMS (Fig. 3D). The statistical comparison confirmed the higher error in the prediction of the EDSS of the SPMS group (*P* = 0.017). We found no significant results when trying to predict the FSS, the SDMT and the BDI.

**Figure 4:**
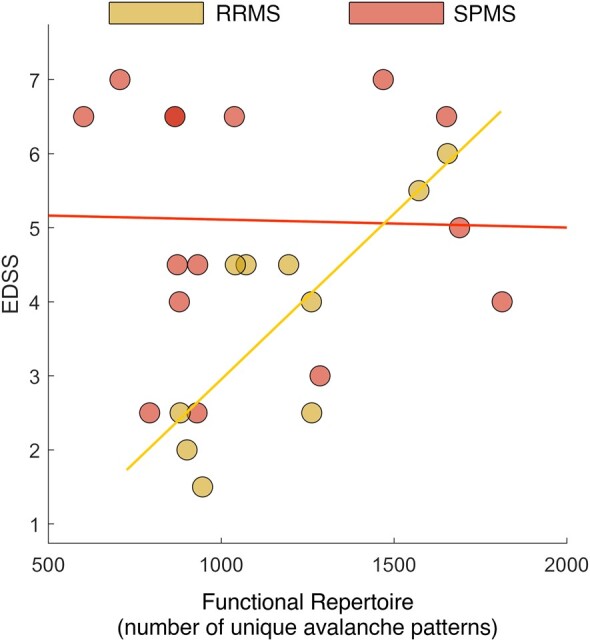
**Correlation between EDSS and brain flexibility**. Spearman correlation test between EDSS and functional repertoire was performed in RRMS and SPMS, separately. Significant correlation was found in the RRMS group only. For SPMS individuals (red points and line), no significant relationship was found between brain flexibility and EDSS (*r* = −0.13, *P* = 0.64). In RRMS subjects (yellow points and line), the functional repertoire was significantly and positively related to the EDSS (*r* = 0.7, *P* = 0.024).

The relationship highlighted by the regression model was confirmed through a Spearman correlation test, performed in each MS group, separately (Fig. 4). For SPMS patients, no significant relationship was found between dynamics and clinical features (*r* = −0.13, *P* = 0.64). Conversely, in RRMS subjects, the functional repertoire was significantly and positively related to the EDSS (*r* = 0.7, *P* = 0.024). Similar results were evident also with the other clinical characteristics, but again, only in the RRMS group. In particular, SDMT and BDI showed respectively a significant negative (*r* = −0.64, *P* = 0.044) and positive (*r* = 0.65, *P* = 0.049) correlation with the size of the functional repertoire. A trend towards a positive statistically significant correlation was found for the fatigue scale. No significant (or nearly significant) relationships between clinical data and brain flexibility were found in the SPMS group.

We also searched for a potential relationship between disease duration and brain flexibility in SPMS, performing a Spearman’s correlation that showed a trend towards a negative correlation between brain dynamics and disease duration (*r* = −0.48, *P* = 0.067).

A multilinear model with the size of the functional repertoire as the dependent variable; age, gender and education as covariates; and each ROI-specific volume as an independent variable has been built. The same model has been used by replacing volume data with LL. In none of the performed models, either LL or volumes had a significant impact on the number of unique brain reconfigurations.

Comparable analyses were performed using clinical markers of active disease (e.g. 2 years with no evidence of disease activity NEDA and the number of clinical relapses) without finding any statistically significant impact of disease activity on the size of the functional repertoire.

We also assessed the impact of the EDSS nonlinearity on our results. To study weather this nonlinearity affects the prediction model and if the relationship between EDSS and functional repertoire was also valid in both lower disability and higher disability, we split the sample into two smaller ones according to the EDSS cut-off of 6. In the cohort with EDSS ≤ 6, we performed a Spearman’s test according to the MS type. A positive correlation was evident in RRMS (*P*-value = 0.02), whereas no relationship was found in the SPMS (*r* = 0.3, *P*-value = 0.4). In the sample with EDSS over 6, composed only of SPMS patients, the absence of a relationship between the functional repertoire size and the EDSS was confirmed. To assess again whether these differences were valid for both lower and higher disability (and not dependent on the particular cut-off value we chose), we also divided the cohort according to the median EDSS value. In both the samples (under and over EDSS of 4.5), we found the same behaviour previously found in the non-split cohort but this time without reaching statistical significance (Supplementary Fig. 2).

## Discussion

In the present study, we investigated the flexibility of the brain dynamics in MS and its relationship with the clinical phenotypes. In particular, following our previous studies carried out on neurodegenerative diseases, including amyotrophic lateral sclerosis,^18^ Alzheimer’s disease^19^ and Parkinson’s disease,^17^ where a reduction of the functional repertoire has been consistently found, we hypothesized that MS could express different behaviours as a function of the prevalence of neuroinflammation or neurodegeneration. When we looked at the different MS clinical phenotypes, we found that the significant differences in the flexibility of the brain dynamics were mainly attributable to the RRMS phenotype. In particular, RRMS patients showed a larger number of unique patterns when compared with HC, whereas the dynamics in SPMS patients did not differ significantly as compared with HC.

In the second part of our work, we searched for relationships between flexibility and MS clinical features. Even in this case, RRMS and SPMS behaved in two opposite ways. The size of the functional repertoire did not show significant predictive power on EDSS. Nevertheless, the interaction between the size of the functional repertoire and MS phenotype showed significant predictive power. That is to say that brain dynamics show different predictive power according to the MS phenotype. In particular, brain dynamics positively correlated with disability in RRMS patients without showing a significant relationship in SPMS patients. This means that only for the RRMS, the increased number of patterns was associated with a worse clinical condition. Accordingly, in the RRMS group, a significant correlation between increased brain flexibility and other clinical signs such as depression and cognitive impairment (assessed by BDI and SDMT, respectively) was also found.

In our opinion, these results could hide two different explanations. On one hand, the observation that only RRMS patients showed larger functional repertoires as compared with HC (and not SPMS patients) could suggest that the increased number of reconfigurations observed in RRMS brain might represent a compensatory mechanism adopted by the nervous system in the early phase of disease to maintain a proper functionality. On the other hand, the difference in brain dynamics between RRMS and HC could be due, at least partially, to the different pathophysiologies that characterizes RRMS and SPMS.

However, the results on the correlation between brain flexibility and clinical condition in RRMS subjects would seem to reject the compensatory hypothesis. In fact, when we performed statistical correlations between brain dynamics and clinical characteristics such as processing speed, depression, fatigue and disability, we found a positive relation between higher brain flexibility and worse clinical condition. All this seems to make a compensatory mechanism a less likely explanation unless the compensatory mechanism is not efficient. On the contrary, the neuropathological differences between the two MS phenotypes could partly explain the diversity in brain flexibility according to the clinical form of the disease. In fact, RRMS is a phenotype with predominant neuroinflammation and demyelination, whereas neurodegeneration, independent from the inflammatory responses, represents the main mechanism of SPMS disease progression.^3,42^ Hence, while the RRMS pathology is dominated by a peripheral immune response (brain parenchymal lymphocyte infiltration through a disrupted blood–brain barrier) that leads to the formation of new active lesions,^43^ progressive MS is characterized by subpial demyelinated lesions, with slow expansion of pre-existing white matter lesions and, most importantly, diffuse grey matter neurodegeneration.^1^ Speculatively, one can suppose that the coexistence of neuroinflammation and neurodegeneration could result in an unchanged level of overall flexibility in SPMS patients. In other words, the similar overall flexibility observed in SPMS with respect to HC may be the result of two concurrent mechanisms that affect the brain dynamics in opposite ways. One pattern would lead to more stereotyped brain dynamics, as previously found in neurodegenerative diseases. A different effect, as seen in the predominantly focal inflammatory RRMS phenotype, would lead to increased heterogeneity of the repertoires. In other words, the disruption of the myelin sheath observed in RRMS would lead to a dysregulation of the overall dynamics, which would become less effectively controlled, resulting in more disordered dynamics and a higher number of states. Widespread degeneration, on the other hand, prevents the brain from accessing certain configurations, which would result in an impoverished repertoire. A comparable amount of both neurodegenerative and focal neuroinflammatory processes might contrast each other.^40^ According to this line of thought, we found a trend towards an inverse correlation between the disease duration and the flexibility of the brain dynamics in SPMS patients. That is to say, the more SPMS patients are close to the early phase of the disease (and, thus, the more similar they are to RRMS), the greater the flexibility of the brain dynamics, while longer disease durations (with increased neurodegenerative load) correspond to impaired flexibility (Supplementary Fig. 1).

Nevertheless, the compensatory hypothesis should not be completely discarded because the possibility of a partial compensation remains viable. Hence, to better interpret and quantify the role of neuroinflammation and neurodegeneration, as well as their impact on the brain dynamics, a larger sample, assessing both neurodegenerative (e.g. neurofilament, Tau and *p*-Tau, and amyloid-β 40/42, in both plasma and CSF) and inflammatory (such as several immune mediators and cytokines) biomarkers, will be necessary in future studies.

Overall, our results are in line with the very recent studies that, through different neuroimaging techniques, investigated brain dynamics in MS. von Schwanenflug *et al*.^44^ conducted an fMRI study in a cohort of almost entirely RRMS subjects, showing an increased flexibility of brain dynamics in MS patients. Similarly, by means of fMRI, Broeders *et al*.^45^ evaluated the brain dynamics in a cohort consisting of around 80% RRMS subjects. They found a greater number of brain network reconfigurations in patients with higher cognitive impairment. These results are in agreement with our findings that show a positive relationship between worse clinical condition and higher brain flexibility in RRMS subjects.

In addition, it is noteworthy that the fatigue burden was significantly different between RRMS and SPMS individuals, and the fact that this difference might have had a role in determining different dynamics between the two groups cannot be ruled out. Although the performed statistical analyses (above all the study of the interaction effect between FSS and MS type in predicting the size of the functional repertoire) do not seem to support this option, wider samples are needed to explore this aspect extensively. The study of the relationship between fatigue and the number of dynamic brain reconfigurations may also help to expand our knowledge about the aetiology of fatigue. Based on the well-known double nature, degenerative and inflammatory,^46^ of fatigue, the positive relationship between FSS score and the number of unique brain reconfigurations in the RRMS and its absence in SPMS could hint at a shift towards a more ‘atrophy-driven’ fatigue along with the disease progression.

Some limitations of the current work should be pointed out. First is the small sample size of the studied population. A second limitation is the absence of CSF/serum inflammatory biomarkers (or neuroinflammatory PET imaging biomarkers) to support the primary role of inflammation in affecting brain dynamics. An adjunctive weakness is the absence of a follow-up of the RRMS patients to demonstrate a change of brain dynamics in case of disease conversion to the progressive form. Future longitudinal studies evaluating the brain flexibility changes across the disease progression (and conversion) will be mandatory to confirm our hypothesis.

To our knowledge, we are the first to investigate brain dynamics through high temporal resolution techniques (M/EEG) in both RRMS and SPMS patients. These findings support the key role of temporal dynamics in understanding the link between brain connectivity and clinical features, also unveiling different dynamical features according to the MS phenotype. Additionally, if the supposed link between brain dynamics, inflammation and clinical outcome will be confirmed, this could allow monitoring in a minimal invasive and objective way (M/EEG analyses) the efficacy of MS immunotherapy.^4,47,48^

## Supplementary Material

fcae112_Supplementary_Data

## Data Availability

The data that support the findings of this study are available from the corresponding author, P.S., upon reasonable request.
