# Supplementary material for: Flexibility of brain dynamics is increased and predicts clinical impairment in relapsing–remitting but not in secondary progressive multiple sclerosis
Source: Brain Commun. 2024 Apr 2;6(2):fcae112. doi: 10.1093/braincomms/fcae112 (PMC10998461; doi:10.1093/braincomms/fcae112)
Supplement: fcae112_Supplementary_Data [file fcae112_supplementary_data.docx]

**Flexibility of brain dynamics is increased and predicts clinical impairment in Relapsing-Remitting but not in Secondary Progressive Multiple Sclerosis**

**SUPPLEMENTARY MATERIAL**

**Supplementary Table 1**

| *Region of Interest* | *RRMS (10)* | *SPMS (13)* | *p-value* |
| --- | --- | --- | --- |
|  |  |  |  |
| Precentral_L | 8.65 ± 1.60 | 7.38 ± 1.17 | ns |
| Precentral_R | 8.21 ± 1.58 | 6.86 ±1.09 | ns |
| Frontal_Sup_L | 7.95 ± 1.58 | 7.59 ±1.08 | ns |
| Frontal_Sup_R | 9.51 ± 1.34 | 8.36 ± 1.38 | ns |
| Frontal_Sup_Orb_L | 2.80 ± 0.50 | 2.47 ± 0.41 | ns |
| Frontal_Sup_Orb_R | 2.96 ± 0.49 | 2.69 ± 0.35 | ns |
| Frontal_Mid_L | 13.38 ± 2.64 | 11.33 ± 1.88 | ns |
| Frontal_Mid_R | 14.39 ± 2.60 | 12.43 ± 1.92 | ns |
| Frontal_Mid_Orb_L | 2.59 ± 0.53 | 2.39 ± 0.38 | ns |
| Frontal_Mid_Orb_R | 3.22 ± 0.47 | 3.03 ± 0.49 | ns |
| Frontal_Inf_Oper_L | 2.83 ± 0.61 | 2.38 ± 0.35 | ns |
| Frontal_Inf_Oper_R | 3.80 ± 0.77 | 3.18 ± 0.42 | ns |
| Frontal_Inf_Tri_L | 6.77 ± 1.30 | 5.90 ± 0.98 | ns |
| Frontal_Inf_Tri_R | 5.21 ± 1.07 | 4.53 ± 0.60 | ns |
| Frontal_Inf_Orb_L | 5.51 ± 0.84 | 4.98 ± 0.70 | ns |
| Frontal_Inf_Orb_R | 5.50 ± 0.91 | 5.01 ± 0.78 | ns |
| Rolandic_Oper_L | 3.03 ± 0.47 | 2.65 ± 0.38 | ns |
| Rolandic_Oper_R | 4.15 ± 0.72 | 3.56 ± 0.41 | ns |
| Supp_Motor_Area_L | 5.36 ± 1.23 | 5.09 ± 0.92 | ns |
| Supp_Motor_Area_R | 5.67 ± 1.13 | 5.16 ± 0.82 | ns |
| Olfactory_L | 0.98 ± 0.16 | 0.92 ± 0.14 | ns |
| Olfactory_R | 0.99 ± 0.17 | 0.95 ± 0.13 | ns |
| Frontal_Sup_Medial_L | 7.30 ± 2.05 | 6.68 ± 1.13 | ns |
| Frontal_Sup_Medial_R | 5.91 ± 1.32 | 5.76 ± 0.79 | ns |
| Frontal_Med_Orb_L | 2.42 ± 0.57 | 2.13 ± 0.38 | ns |
| Frontal_Med_Orb_R | 2.87 ± 0.59 | 2.56 ± 0.39 | ns |
| Rectus_L | 2.63 ± 0.54 | 2.28 ± 0.35 | ns |
| Rectus_R | 2.13 ± 0.43 | 1.91 ± 0.22 | ns |
| Insula_L | 6.60 ± 1.11 | 6.17 ± 0.88 | ns |
| Insula_R | 6.41 ± 1.06 | 5.87 ± 0.88 | ns |
| Cingulum_Ant_L | 4.71 ± 0.98 | 4.23 ± 0.68 | ns |
| Cingulum_Ant_R | 4.25 ± 0.87 | 3.89 ± 0.63 | ns |
| Cingulum_Mid_L | 6.17 ± 1.28 | 5.35 ± 1.10 | ns |
| Cingulum_Mid_R | 6.83 ± 1.42 | 6.17 ± 0.77 | ns |
| Cingulum_Post_L | 1.17 ± 0.21 | 1.03 ± 0.17 | ns |
| Cingulum_Post_R | 0.59 ± 0.10 | 0.55 ± 0.07 | ns |
| Hippocampus_L | 3.61 ± 0.36 | 3.18 ± 0.44 | ns |
| Hippocampus_R | 3.34 ± 0.27 | 3.03 ± 0.38 | ns |
| ParaHippocampal_L | 3.47 ± 0.37 | 3.31 ± 0.37 | ns |
| ParaHippocampal_R | 4.20 ± 0.45 | 3.97 ± 0.49 | ns |
| Amygdala_L | 0.97 ± 0.15 | 0.87 ± 0.11 | ns |
| Amygdala_R | 1.00 ± 0.14 | 0.96 ± 0.11 | ns |
| Calcarine_L | 7.36 ± 1.08 | 6.35 ± 1.17 | ns |
| Calcarine_R | 5.20 ± 0.82 | 4.51 ± 1.01 | ns |
| Cuneus_L | 4.17 ± 0.74 | 3.66 ± 0.47 | ns |
| Cuneus_R | 3.87 ± 0.60 | 3.30 ± 0.55 | ns |
| Lingual_L | 6.61 ± 0.85 | 5.83 ± 0.81 | ns |
| Lingual_R | 6.73 ± 1.04 | 5.93 ± 0.79 | ns |
| Occipital_Sup_L | 3.15 ± 0.55 | 2.81 ± 0.36 | ns |
| Occipital_Sup_R | 3.39 ± 0.50 | 3.00 ± 0.38 | ns |
| Occipital_Mid_L | 9.09 ± 1.57 | 8.14 ± 1.08 | ns |
| Occipital_Mid_R | 6.47 ± 0.97 | 5.86 ± 0.83 | ns |
| Occipital_Inf_L | 2.54 ± 0.39 | 2.26 ± 0.41 | ns |
| Occipital_Inf_R | 3.07 ± 0.48 | 2.68 ± 0.43 | ns |
| Fusiform_L | 9.03 ± 1.04 | 8.21 ± 1.11 | ns |
| Fusiform_R | 9.95 ±1.26 | 9.00 ± 1.10 | ns |
| Postcentral_L | 9.31 ± 1.25 | 7.99 ± 1.19 | ns |
| Postcentral_R | 9.26 ± 1.32 | 8.13 ± 1.16 | ns |
| Parietal_Sup_L | 4.92 ± 0.68 | 4.47 ± 0.72 | ns |
| Parietal_Sup_R | 5.01 ± 0.67 | 4.56 ± 0.73 | ns |
| Parietal_Inf_L | 7.02 ± 1.31 | 6.18 ± 1.02 | ns |
| Parietal_Inf_R | 4.33 ± 0.82 | 3.94 ± 0.47 | ns |
| SupraMarginal_L | 3.32 ± 0.57 | 2.89 ± 0.35 | ns |
| SupraMarginal_R | 5.79 ± 1.01 | 5.09 ± 0.56 | ns |
| Angular_L | 3.23 ± 0.74 | 2.97 ± 0.50 | ns |
| Angular_R | 5.27 ± 0.96 | 4.90 ± 0.95 | ns |
| Precuneus_L | 9.78 ± 1.52 | 8.57 ± 1.12 | ns |
| Precuneus_R | 8.71 ± 1.35 | 7.82 ± 1.13 | ns |
| Paracentral_Lobule_L | 2.71 ± 0.56 | 2.39 ± 0.46 | ns |
| Paracentral_Lobule_R | 1.68 ± 0.36 | 1.47 ± 0.23 | ns |
| Caudate_L | 3.20 ± 0.76 | 2.66 ± 0.78 | ns |
| Caudate_R | 3.44 ± 0.95 | 3.04 ± 0.80 | ns |
| Putamen_L | 3.54 ± 1.08 | 2.94 ± 0.74 | ns |
| Putamen_R | 3.22 ± 1.01 | 2.99 ± 0.66 | ns |
| Pallidum_L | 0.33 ± 0.15 | 0.25 ± 0.08 | ns |
| Pallidum_R | 0.32 ± 0.13 | 0.28 ± 0.11 | ns |
| Thalamus_L | 3.34 ± 1.10 | 2.69 ± 0.93 | ns |
| Thalamus_R | 3.29 ± 1.26 | 2.89 ± 0.96 | ns |
| Heschl_L | 0.72 ± 0.17 | 0.62 ± 0.15 | ns |
| Heschl_R | 0.82 ± 0.18 | 0.66 ± 0.15 | ns |
| Temporal_Sup_L | 7.20 ± 1.31 | 6.22 ± 1.09 | ns |
| Temporal_Sup_R | 9.34 ± 1.47 | 8.08 ± 1.08 | ns |
| Temporal_Pole_Sup_L | 3.64 ± 0.78 | 3.19 ± 0.57 | ns |
| Temporal_Pole_Sup_R | 3.66 ± 0.62 | 3.42 ± 0.49 | ns |
| Temporal_Mid_L | 15.06 ± 3.05 | 13.60 ± 1.68 | ns |
| Temporal_Mid_R | 14.93 ± 2.46 | 13.39 ± 1.96 | ns |
| Temporal_Pole_Mid_L | 2.70 ± 0.36 | 2.50 ± 0.32 | ns |
| Temporal_Pole_Mid_R | 3.39 ± 0.42 | 3.12 ± 0.39 | ns |
| Temporal_Inf_L | 10.94 ± 1.99 | 9.97 ± 1.12 | ns |
| Temporal_Inf_R | 12.79 ± 1.89 | 11.49 ± 1.43 | ns |

Supplementary Table 1. *ROI volumes of MS patients according to the clinical form*. p-value from Wilcoxon-Mann-Whitney or Student's t-test, according to the sample data distribution checked with the Kolmogorov-Smirnov test. Following adjusting for multiple testing (False discovery rate correction) no differences were found between the two groups. Volumes are defined in mL.

Abbreviations: Ant: anterior; L: left; Oper: opercular; Orb: orbital; Mid: middle; Inf: inferior; Post: posterior; R:right; Sup: superior; Supp: supplementary; Tri: triangular.

**Relationship between disease duration and functional repertoire according to the MS type**.

**Supplementary Figure 1**


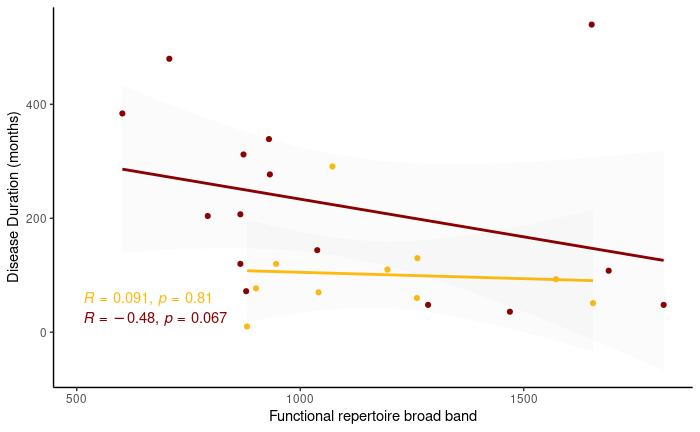


**Supplementary Figure 1**. **Functional repertoire and disease duration relationship**. Spearman’s correlation between functional repertoire and disease duration for each MS phenotype (RRMS in yellow; SPMS in red). No significant correlations were found in both RRMS (R=0.091; p-value=0.81) and SPMS patients (R=-0.48; p-value=0.067).

These results suggest effects of the temporal relation between RRMS and SPMS on the functional repertoire size. SPMS seems to have greater variability in the size of functional repertoire. This difference could be explained by the combination of the two underlying pathological mechanisms of the SPMS and by the fact that the entity of the relative impact on the disease evolves and changes over time and disease subtypes (e.g. neurodegeneration increases over time). This concept is supported by a negative correlation (p-value 0.067) between disease duration and the size of the functional repertoire in SPMS. The more SPMS is temporally near to the RRMS, the larger the functional repertoire (i.e. the more similar to the RRMS patients). Conversely, the more SPMS patients are temporally distant from the RRMS phase, the smaller the functional repertoire. The combination of the two pathological mechanisms in the SPMS could determine greater variability in the size of the functional repertoire.

**Effects of the EDSS nonlinearity**.

EDSS is nonlinear: higher scores reflect more severe disability. To study weather this nonlinearity affects the prediction model and if the relationship between EDSS and functional repertoire is also valid in both lower disability and higher disability, we split the sample into two smaller ones according to the EDSS cut-off of 6. In the cohort with EDSS ≤ 6, we performed a Spearman’s test according to the MS type. A positive correlation was evident in RRMS (p-value=0.02) whereas no relationship was found in the SPMS (r=0.3, p-value=0.4). In the sample with EDSS over 6, composed only of SPMS patients, the absence of a relationship between the functional repertoire size and the EDSS was confirmed. To assess again whether these differences were valid for both lower and higher disability (and not dependent on the particular cut-off value we chose), we also divided the cohort according to the median EDSS value. In both the samples (under and over EDSS of 4.5) we found the same behaviour previously found in the non-split cohort, but this time without reaching statistical significance (see the figure below for the more uniform, 7 RRMS and 7 SPMS, sample of EDSS under 4.5).

**Supplementary Figure 2**


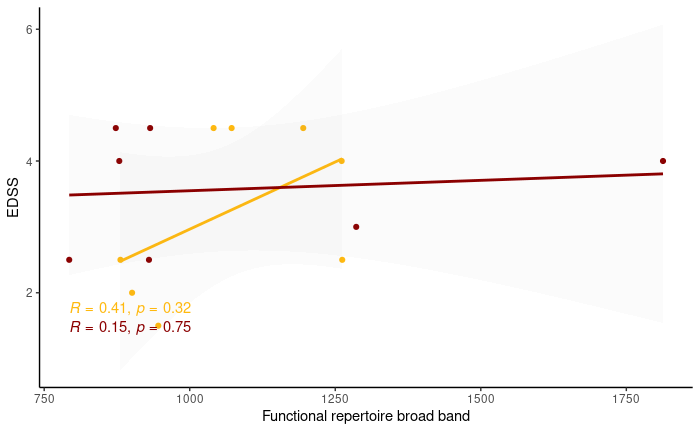


**Supplementary Figure 2**. **Functional repertoire and EDSS relationship**. Spearman’s correlation between functional repertoire in the broad band and EDSS according to the MS form (RRMS in yellow; SPMS in red). No significant correlations were found in both RRMS (R=0.41; p-value=0.32) and SPMS patients (R=0.15; p-value=0.75).Abbreviations: Expanded Disability Status Scale (EDSS)
